# Insights into bifunctional active sites of Pt–MoO_3_/TiO_2_ catalysts enabling selective hydrogenation of an amino acid

**DOI:** 10.1039/d5sc05792b

**Published:** 2025-10-09

**Authors:** Yundao Jing, Xiaohu Ge, Rui Song, Ningchao Zhu, Jinquan Ming, Nihong An, Yueqiang Cao, Gang Qian, Xuezhi Duan, Xinggui Zhou

**Affiliations:** a State Key Laboratory of Chemical Engineering and Low-Carbon Technology, School of Chemical Engineering, East China University of Science and Technology 130 Meilong Road Shanghai 200237 China yqcao@ecust.edu.cn xzduan@ecust.edu.cn; b Sino-Platinum Industrial Catalyst (Yunnan) Co., Ltd. 988 Keji Road Kunming 650106 Yunnan China

## Abstract

Selective hydrogenation of amino acids to amino alcohols is a valuable transformation in the synthesis of pharmaceuticals, fine chemicals, and chiral building blocks. However, achieving high activity and selectivity under mild conditions remains challenging due to the need for simultaneous hydrogen activation and substrate coordination. Here, we report a series of Pt–MoO_3_ bifunctional catalysts for the hydrogenation of l-alanine (Ala) to alaninol (AlaOH), with a focus on tuning metal–oxide synergy. Structural and electronic characterization studies by high-angle annular dark-field scanning transmission electron microscopy, X-ray photoelectron spectroscopy and X-ray absorption spectroscopy reveal strong Pt–MoO_3_ interactions, characterized by partial electron transfer. Catalytic tests reveal a volcano-type dependence on the Pt/Mo ratio, with the 4-Pt–MoO_3_ catalyst achieving the highest performance. The experiments of H_2_ temperature programmed desorption and *in situ* diffuse reflectance infrared Fourier transform spectroscopy combined with theoretical calculations support a bifunctional mechanism, in which Pt serves as the primary site for H_2_ activation, while MoO_3_ facilitates adsorption and stabilization of polar alanine. Further tuning *via* thermal treatments shows that the moderate treatment at 500 °C optimally balances the redox state of MoO_3_ without compromising Pt dispersion, leading to enhanced hydrogenation performance. This work not only advances understanding of metal–oxide interfacial catalysis but also provides a rational design strategy for efficient and selective hydrogenation of amino acids.

## Abstract 2

A bifunctional Pt–MoO_3_ catalyst enables selective amino acid hydrogenation *via* tunable interfacial synergy, achieved through control of composition and the redox state.

## Graphical abstract

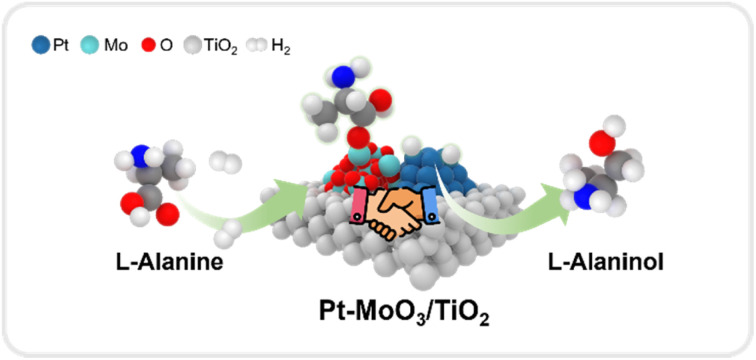

## 1 Introduction

Transformation of amino acids into value-added amino alcohols is an important process for the synthesis of chiral intermediates, pharmaceutical building blocks and nitrogen-containing fine chemicals.^1–4^ However, conventional organic synthesis of amino alcohols from amino acids remains challenging due to the strong polarity of carboxyl and amino functional groups, which often results in the formation of undesired products from side reactions such as deamination or over-reduction under harsh conditions.^5–9^ To overcome these limitations, the development of high-performance catalysts that enable selective hydrogenation under mild conditions is highly desirable.^10–12^ Although catalytic hydrogenation offers a more efficient and sustainable alternative to conventional organic synthesis, such a process normally requires elevated hydrogen pressures (>6 MPa), under which by-products such as amines are often readily formed alongside the desired amino alcohols.^13,14^ Recently, bifunctional catalysts composed of both metal and metal oxide components, which are capable of coupling hydrogen activation with substrate adsorption, have emerged as a promising strategy to achieve efficient and selective hydrogenation under mild reaction conditions.^15–18^ Building on these pioneering studies in the hydrogenation of other substrates, to design a bifunctional catalytic system would be promising for enhancing the catalytic performance for the hydrogenation of amino acids.

Among metal–oxide bifunctional systems, the metal sites, such as Pd and Pt sites, typically facilitate the dissociation and activation of hydrogen molecules, while the metal oxide components contribute to the adsorption and activation of polar substrates, such as carbonyl-, carboxyl- and nitro-containing molecules.^19,20^ A representative example is the Pt_1_Al_1_/MgO catalyst, in which highly dispersed Pt sites confined on the MgO(100) surface facilely activate H_2_ to generate reactive hydrogen species. Such species then migrate to the Al–O–Pt moieties on MgO to hydrogenate the polar substrates adsorbed there, demonstrating a clear synergy between metal sites and oxide sites for catalytic hydrogenation of 3-nitrostyrene.^16,21^ Another example is the Pt/CeO_2_ catalyst, where Pt clusters activate H_2_ to form reactive hydrogen species that spillover to hydrogenate nitro groups of the substrate bound to CeO_2_ vacancies, also highlighting the cooperative roles of metal and metal oxide in the hydrogenation of the substrate.^15^ Similarly, on the Pt–MoO_*x*_ bifunctional catalyst, Pt supplies activated H_2_, while Mo vacancy sites bind and polarize the acyl carbonyl, enabling a reverse Mars–van Krevelen hydrodeoxygenation (HDO) pathway toward ethers. Consistent with such a bifunctional paradigm, the Pt–Mo/ZrO_2_ system achieves high ether selectivity under remarkably mild conditions,^22^ and a complementary sulfoxide HDO study likewise evidences this Pt–MoO_*x*_ cooperation.^23^ In spite of these bifunctional systems well designed for such successful cases, mechanistic understanding of the hydrogenation of amino acids, particularly with respect to the nature and function of active sites, has not been clearly revealed but remains crucial for the rational design of high-performance catalysts.^24^

By choosing Pt and Mo oxides as the corresponding metal and metal oxides, we hereby design a series of Pt–MoO_3_/TiO_2_ catalysts with varied Pt/Mo ratios supported on rutile TiO_2_ to explore the synergistic roles of metal–oxide sites in the hydrogenation of amino acids exemplified with l-alanine. The morphologies and detailed structures of the catalysts were characterized by multiple techniques, such as high-angle annular dark-field scanning transmission electron microscopy (HAADF-STEM), X-ray photoelectron spectroscopy (XPS) and X-ray absorption spectroscopy analysis. The results reveal the occurrence of electron transfer from Pt to MoO_3_, which promotes the formation of MoO_*x*_ species composed of lower-valence Mo species and oxygen vacancies. Hydrogen temperature-programmed desorption (H_2_-TPD) and *in situ* diffuse reflectance infrared Fourier transform spectroscopy (*in situ* DRIFTS) studies demonstrate that the Pt sites dominate the dissociative activation of hydrogen while the partially reduced MoO_3_ acts as the anchoring site for carboxyl groups of l-alanine (Ala). Furthermore, density functional theory (DFT) calculations were also employed to rationalize such synergy between Pt and MoO_3_ species for the hydrogenation of Ala. We finally demonstrate that both the compositional balance and thermal pretreatment govern the synergy of Pt and MoO_3_, with optimal hydrogenation activity achieved at a specific Pt/Mo ratio and reduction temperature. This work provides fundamental insight into interface-driven catalysis and establishes a generalizable strategy for the rational design of selective hydrogenation catalysts for amino acids and even other polar substrates.

## 2 Experiments

### Synthesis of Pt–MoO_3_/TiO_2_

The Pt–MoO_3_/TiO_2_ catalysts with varying Pt/Mo atomic ratios were synthesized *via* a sequential incipient wetness impregnation method. Initially, Pt/TiO_2_ was prepared by impregnating commercial titanium dioxide (TiO_2_, >99%, *P*4_2_/*mnm*, Aladdin, China) with an aqueous solution of chloroplatinic acid (H_2_PtCl_6_·6H_2_O, >99.5%, Macklin, China), followed by aging at room temperature for 12 h, drying at 80 °C overnight, and calcination at 400 °C for 4 h in air. Then, ammonium molybdate ([(NH_4_)_6_Mo_7_O_24_·4H_2_O], >99.5%, Macklin, China) was employed as the molybdenum precursor to introduce Mo onto the pre-synthesized Pt/TiO_2_ by a second impregnation step. By adjusting the concentration of the molybdate solution, Pt/Mo atomic ratios of 6 : 1, 4 : 1, 2 : 1 and 1 : 1 were obtained. The impregnated samples were aged, dried at 80 °C, ground, and calcined at 400 °C for 4 h to afford the final Pt–MoO_3_/TiO_2_ catalysts. All catalysts were prepared with a fixed Pt loading of 5 wt%. The resulting catalysts were denoted as 6-Pt–MoO_3_, 4-Pt–MoO_3_, 2-Pt–MoO_3_, and 1-Pt–MoO_3_, respectively, where the leading numbers indicate the Pt/Mo atomic ratio.

### Catalytic performance evaluation

The catalytic activity was evaluated *via* the hydrogenation of Ala to AlaOH, which was carried out in a stainless-steel autoclave equipped with a 100 mL polytetrafluoroethylene liner. Typically, 30 mL of Ala aqueous solution was transferred into the reactor liner, followed by the addition of 100 mg catalyst and 450 μL 85 wt% phosphoric acid. After sealing, the autoclave was purged several times with high-purity argon to remove residual air, and then heated to 95 °C. Unless otherwise noted, a small aliquot (450 μL) of 85 wt% H_3_PO_4_ was added to protonate Ala,^25^ to stabilize the amino acid form,^26^ and to provide a mild, reproducible proton environment that accelerates hydrogenation kinetics. It should be noted that phosphate species are known to bind strongly to TiO_2_ and can become irreversibly grafted upon drying/calcination.^27^ However, under these liquid-phase reaction conditions at 95 °C, no dehydration or calcination pretreatment is involved. Furthermore, *in situ* AcOH-DRIFTS and catalytic tests indicated that TiO_2_ is not the primary active site for the hydrogenation and the introduced MoO_*x*_ provides the principal –COOH adsorption/activation sites. Therefore, it is reasonable to believe that any transient adsorption of phosphate on TiO_2_, if present under our mild liquid-phase conditions, would have limited impact on the catalytic performance of Pt–MoO_3_/TiO_2_ bifunctional catalysts. Once the temperature stabilized, the reactor was purged and charged with high-purity hydrogen (>99.999%) to 4 MPa, and stirring was initiated at 800 rpm to commence the reaction. Upon completion, 1 mL of the reaction mixture was collected, filtered through a 0.22 μm syringe filter, and analyzed using ultra-performance liquid chromatography (UPLC, Waters Acquity). A ZORBAX SB-C18 column (4.6 mm × 150 mm, 5 μm) coupled with a photodiode array detector was employed. Since both Ala and its hydrogenation product, AlaOH, exhibit weak ultraviolet absorption in the range of 190–400 nm, derivatization with phenyl isothiocyanate (PITC) of the reaction solution was performed prior to analysis to enhance their UV detectability.^28^

## 3 Results and discussion

### Morphologies and structures of bifunctional Pt–MoO_3_/TiO_2_ catalysts

**Fig. 1:**
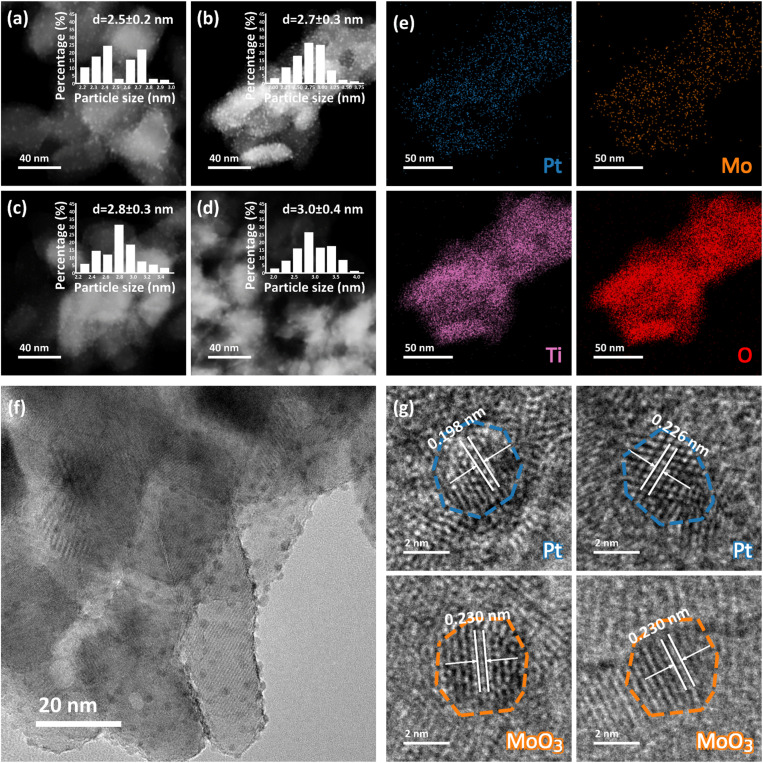
HAADF-STEM images and corresponding particle size distributions of the (a) 6-Pt–MoO_3_, (b) 4-Pt–MoO_3_, (c) 2-Pt–MoO_3_ and (d) 1-Pt–MoO_3_ catalysts. (e) HAADF-STEM EDS mapping images, (f) TEM image and (g) HRTEM images of the 4-Pt–MoO_3_ catalyst.

The morphologies and structures of the as-synthesized Pt–MoO_3_/TiO_2_ bifunctional catalysts with different Pt/Mo atomic ratios were characterized by multiple techniques. Fig. 1a–d show the HAADF-STEM images of 6-Pt–MoO_3_, 4-Pt–MoO_3_, 2-Pt–MoO_3_, and 1-Pt–MoO_3_ catalysts, respectively, where the initial numbers denote the Pt/Mo ratio. As clearly seen in these HAADF-STEM images, the supported nanoparticles are well dispersed on TiO_2_ with no obvious aggregation for all the samples. The particle size distributions were obtained by analyzing over 200 nanoparticles in each sample from the collected HAADF-STEM images. The determined average particle sizes for 6-Pt–MoO_3_, 4-Pt–MoO_3_, 2-Pt–MoO_3_, and 1-Pt–MoO_3_ catalysts are 2.5 ± 0.2, 2.7 ± 0.3, 2.8 ± 0.3 and 3.0 ± 0.4 nm, respectively, showing similar particle sizes with narrow size distributions. These results demonstrate similar morphologies for these Pt–MoO_3_/TiO_2_ catalysts with varied Pt/Mo atomic ratios.

HAADF-STEM EDS mapping analysis was further employed to determine the structures of catalysts. As illustrated in Fig. 1e, HAADF-STEM elemental mapping reveals a highly uniform distribution of Pt, Mo, Ti, and O throughout the 4-Pt–MoO_3_ catalyst. The clear spatial overlap between Pt and Mo signals indicates a close proximity between these two active species, which is critical for promoting synergistic effects at the nanoscale. Complementary transmission electron microscopy (TEM) and high-resolution TEM (HRTEM) images further support the uniform dispersion of nanoparticles across the support (Fig. 1f), indicating effective control over particle size and distribution. The HRTEM images offer insights into the crystalline nature of the catalyst (Fig. 1g). Lattice fringes with measured spacings of 0.196 nm and 0.226 nm can be assigned to the (200) and (111) planes of metallic Pt, respectively (JCPDS no. 87-0646). Meanwhile, the 0.230 nm of fringe spacing corresponds to the (060) plane of orthorhombic MoO_3_ (JCPDS no. 01-0706). The coexistence of well-defined lattice fringes from both components within the same field of view further confirms the structural integration of Pt and MoO_3_ at the nanoscale. The combination of HAADF-STEM and HRTEM analyses not only verifies the compositional uniformity of the catalyst but also unveils the intimate contact that underlies its bifunctional catalytic performance.

**Fig. 2:**
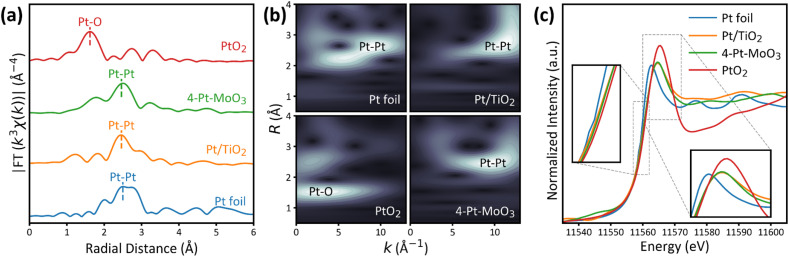
(a) Fourier-transformed *k*^3^-weighted Pt L_3_-edge EXAFS spectra in *R*-space, (b) corresponding WT-EXAFS contour plots, and (c) normalized XANES spectra of the PtO_2_, Pt foil, Pt/TiO_2_ and 4-Pt–MoO_3_ catalysts.

To further probe the detailed structures of Pt–MoO_3_/TiO_2_ bifunctional catalysts, X-ray absorption spectroscopy measurements were also performed for the selected 4-Pt–MoO_3_ catalyst, together with Pt foil, monometallic Pt and PtO_2_ samples as the reference. As illustrated in Fig. 2a, the Fourier-transformed extended X-ray absorption fine structure (FT-EXAFS) spectrum at the Pt L_3_-edge of the Pt foil shows a dominant peak at around 2.4 Å corresponding to Pt–Pt coordination, while that of the PtO_2_ sample exhibits a distinct peak at around 1.6 Å corresponding to Pt–O coordination. For Pt/TiO_2_ and 4-Pt–MoO_3_ catalysts, the FT-EXAFS spectra show main coordination peaks at around 2.6 Å, indicating the presence of metallic Pt–Pt bonding.^29^ Notably, a minor shoulder peak is seen near the Pt–Pt coordination peak in the spectrum of the 4-Pt–MoO_3_ catalyst, pointing to the Pt–Mo coordination in the bifunctional catalyst. This is further supported by the wavelet transformed EXAFS (WT-EXAFS) contour plots in Fig. 2b. The WT contour of PtO_2_ exhibits a strong intensity at low *k* values (*ca.* 3–5 Å^−1^), which is characteristic of Pt–O scattering, as oxygen is a light element (*Z* = 8) that contributes primarily to low-*k* components due to its weak backscattering ability. Such a signal is hardly seen in the contour plots of the Pt foil, Pt/TiO_2_ and 4-Pt–MoO_3_ catalysts, indicating the absence of oxidized Pt species. The reduced coordination number and enhanced quantum confinement in small Pt clusters lead to sharpening of the photoelectron wave packet and suppression of low-*k* scattering paths. More importantly, the scattering center of the contour plot for the 4-Pt–MoO_3_ catalyst shifts slightly toward lower *k* values, as compared to those of the Pt foil and monometallic Pt/TiO_2_ sample. Such a shift could be attributed to the presence of minor Pt–Mo coordination,^30–32^ which is reasonably caused by the intimate contact of Pt and MoO_3_ demonstrated by HAADF-STEM and HRTEM images.

The structural indications of the above-observed Pt–MoO_3_ interaction are further supported by X-ray absorption near-edge structure (XANES) and XPS tests. The Pt L_3_-edge XANES spectra in Fig. 2c reveal distinct variations in the electronic structure of Pt across the catalyst series. As expected, the spectrum of the PtO_2_ reference exhibits a pronounced white-line intensity and a positively shifted absorption edge, characteristic of a high oxidation state close to Pt^4+^. In contrast, that of the Pt foil shows the lowest white-line intensity and an unshifted edge, consistent with a fully metallic Pt^0^ state. The spectra of Pt/TiO_2_ and 4-Pt–MoO_3_ both present slightly elevated white-line intensities and marginally shifted absorption edges relative to Pt foil, suggesting that Pt in these samples remains predominantly metallic.^33^ Notably, the incorporation of MoO_3_ results in a subtle but discernible shift in the absorption edge of 4-Pt–MoO_3_ toward higher energy compared to Pt/TiO_2_. This delayed edge position suggests a minor increase in the average oxidation state of Pt, pointing to electron transfer from Pt to adjacent MoO_3_ species.

**Fig. 3:**
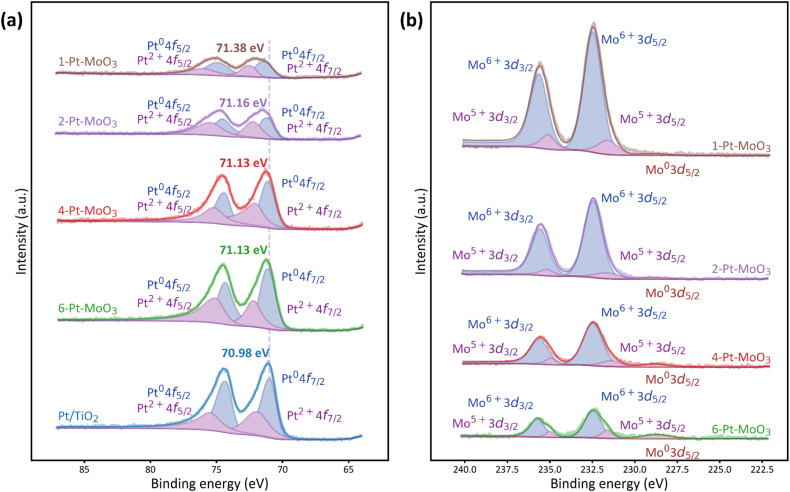
(a) Pt 4f and (b) Mo 3d XPS spectra of the 6-Pt–MoO_3_, 4-Pt–MoO_3_, 2-Pt–MoO_3_ and 1-Pt–MoO_3_ catalysts.

The XPS results further substantiate the XANES findings. In the Pt 4f region (Fig. 3a), all catalysts exhibit the characteristic doublet peaks corresponding to Pt^0^ and Pt^2+^ species.^34–36^ The XPS spectrum of Pt/TiO_2_ displays a dominant Pt^0^ peak at a binding energy of 70.89 eV, reaffirming the metallic nature of Pt in the absence of MoO_3_. However, upon MoO_3_ addition, a progressive positive shift in the Pt 4f_7/2_ binding energy is observed, from 71.13 eV for 6-Pt–MoO_3_ to 71.38 eV for 1-Pt–MoO_3_.

Concurrently, the contribution of Pt^2+^ species becomes increasingly pronounced at higher Mo contents (*i.e.*, lower Pt/Mo ratios). In parallel, the Mo 3d XPS spectra provide insights into the evolution of electronic properties of Mo species in the presence of Pt.^37–39^ All Mo-containing samples predominantly exhibit Mo^6+^ signals, with minor contributions from Mo^5+^ due to the partial reduction of MoO_3_ (Fig. 3b). Interestingly, the Mo 3d XPS spectra of the 4-Pt–MoO_3_ and 6-Pt–MoO_3_ catalysts with high Pt content even show a weak signal assigned to the Mo^0^ component, indicative of complete reduction of minor MoO_3_ to Mo^0^ on the surface, which is likely facilitated by the presence of Pt. Such Pt-induced reduction of MoO_3_ is thought to generate oxygen vacancies on the MoO_3_ surface, which can act as active sites for reactant adsorption, such as amino acid species.^40–42^ Furthermore, as the Mo content increases, the intensity of reduced Mo^5+^ and Mo^0^ features diminishes, implying that excess MoO_3_ buffers the extent of Pt-induced reduction, likely due to electron dilution and spatial separation effects at higher Mo loadings. Together, the XANES and XPS results offer compelling evidence of electronic interactions between Pt and MoO_3_, which is also evidenced by Bader charge analysis. The theoretical results show clear electron depletion on interfacial Pt and accumulation on Mo/O of MoO_3_ (Fig. S29), indicating the interfacial charge transfer.^43,44^

### Bifunctional Pt–MoO_3_/TiO_2_ catalysts for l-alanine hydrogenation

**Fig. 4:**
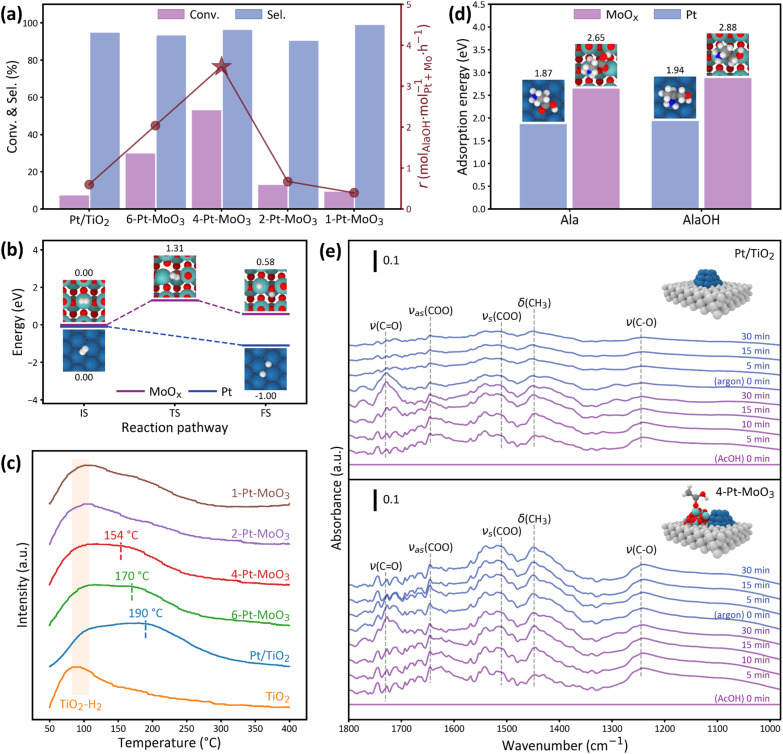
(a) Catalytic performance of Pt–MoO_3_/TiO_2_ catalysts with varying Pt/Mo ratios for Ala hydrogenation: the conversion (purple bars), AlaOH selectivity (blue bars), and reaction rate (red line). (b) Reaction energy profiles for H_2_ dissociation and adsorption on Pt (blue) and MoO_3_ (purple) surfaces. (c) H_2_-TPD profiles of bifunctional Pt–MoO_3_/TiO_2_ and Pt/TiO_2_ catalysts. (d) Calculated adsorption energies of Ala and AlaOH and (e) *in situ* AcOH-DRIFTS spectra of Pt/TiO_2_ and 4-Pt–MoO_3_ catalysts.

Having identified structures of the bifunctional Pt–MoO_3_/TiO_2_ catalysts, we turned our attention to investigate the performance of these catalysts for the hydrogenation of amino acids exemplified with Ala here. As shown in Fig. 4a, the monometallic Pt/TiO_2_ catalyst exhibits low activity, with an Ala conversion below 10%, despite retaining relatively high selectivity toward AlaOH. Upon incorporation of MoO_3_, the catalytic activity is markedly enhanced. Among all catalysts, the 4-Pt–MoO_3_ catalyst demonstrates the highest performance, achieving 53.1% of Ala conversion with 96.3% of AlaOH selectivity. The corresponding formation rate of AlaOH reaches 3.61 mol_AlaOH_ per mol_Pt+Mo_·per h, representing an order of magnitude improvement over the Pt/TiO_2_ catalyst (0.63 mol_AlaOH_ per mol_Pt+Mo_·per h). As the Pt/Mo ratio decreases from 4 to 2 and 1, *i.e.*, the 2-Pt–MoO_3_ and 1-Pt–MoO_3_ catalysts, the catalytic activity in terms of both Ala conversion and AlaOH formation rate declines sharply, leading to a volcano-type dependence on the Pt/Mo ratio. Notably, while the catalytic activity decreases sharply at higher MoO_3_ contents, the selectivity remains consistently high across all catalysts, suggesting that product distribution is largely insensitive to the Pt/Mo ratio.

While the experimental data clearly suggest that the incorporation of MoO_3_ boosts catalytic activity without compromising selectivity, the precise roles of Pt and MoO_3_ in the hydrogenation process remain to be clarified. In particular, whether MoO_3_ directly contributes to hydrogen activation, or instead plays a supporting role in substrate adsorption or intermediate stabilization, is a key question. To address this, a combination of DFT calculations, H_2_-TPD, and *in situ* AcOH-DRIFTS tests was employed. Based on Wulff constructions (Fig. S30, Tables S3 and S4), Pt(111) and MoO_3_(060) were selected as the most thermodynamically stable and experimentally relevant surfaces for DFT studies. The DFT-calculated reaction energy profiles reveal distinct differences in hydrogen activation across these two components (Fig. 4b). On the Pt(111) surface, H_2_ dissociation occurs spontaneously, with a substantial exothermic energy change from 0 to −0.99 eV, indicating the facile formation of active hydrogen species. In contrast, the MoO_*x*_ surface exhibits an energetically unfavorable H_2_ activation pathway, with a high barrier of 1.31 eV and a slightly endothermic adsorption energy (+0.58 eV), confirming that MoO_*x*_ is not intrinsically active for H_2_ activation. These findings indicate that Pt serves as the principal site for hydrogen activation, supplying reactive hydrogen atoms for subsequent hydrogenation steps. H_2_-TPD was also conducted to investigate hydrogen adsorption behavior. As shown in Fig. 4c, the profile of the pure TiO_2_ support exhibits a weak and broad desorption feature centered at around 100 °C, which likely originates from weakly physisorbed hydrogen species. In contrast, the Pt/TiO_2_ catalyst shows a distinct desorption peak centered at 190 °C, which can be assigned to the recombinative desorption of dissociated hydrogen from Pt surface sites. Upon incorporation of MoO_3_, the desorption temperature systematically shifts toward lower values. Specifically, the profiles of 6-Pt–MoO_3_ and 4-Pt–MoO_3_ catalysts exhibit desorption peaks at approximately 170 °C and 154 °C, respectively, indicating progressively weakened hydrogen adsorption strength. For the 2-Pt–MoO_3_ and 1-Pt–MoO_3_ catalysts, the desorption features become broader and less defined, and no clear peak maxima are observed, suggesting a substantial suppression of hydrogen uptake. This downward shift in desorption temperature with increasing MoO_3_ content implies that the interaction between hydrogen and Pt is modulated by the presence of adjacent MoO_3_ species. A plausible explanation is that electronic perturbation of Pt caused by MoO_3_ alters the hydrogen binding energy, consistent with the increased Pt oxidation state determined by XPS and XANES tests. Collectively, these results indicate that while Pt remains the primary site for hydrogen activation, excessive MoO_3_ incorporation adversely affects its function by weakening hydrogen adsorption. This finding correlates well with the observed volcano-type activity trend, highlighting the need for an optimal balance between Pt and MoO_3_ to achieve efficient hydrogen activation and catalytic performance.

To further elucidate the role of the catalyst in substrate activation and product stabilization, DFT calculations were carried out to evaluate the adsorption energies of Ala and its hydrogenation product AlaOH on Pt and MoO_*x*_ surfaces. As shown in Fig. 4d, both molecules exhibit significantly stronger adsorption on MoO_3_ compared to Pt. Specifically, the adsorption energy of Ala on MoO_*x*_ reaches −2.56 eV, while that on Pt(111) is only −1.87 eV. Similarly, AlaOH adsorbs with −2.88 eV on MoO_*x*_*versus* −1.94 eV on Pt(111). These results suggest that MoO_3_ provides a more favourable surface for anchoring polar functional groups such as carboxyl (–COOH) and hydroxyl (–OH), likely due to its electron-deficient metal centers and oxophilic nature. In contrast, the relatively weaker adsorption on Pt implies limited interaction with the polar reactants and products. This mechanistic insight aligns well with the experimental observation that monometallic Pt/TiO_2_ exhibits very low activity (<10% conversion) despite retaining high selectivity, indicating that Pt alone is insufficient to activate the substrate effectively. This preferential adsorption on MoO_3_ is likely to enhance substrate activation and stabilize key intermediates or products, which in turn contributes to the high selectivity observed in experiments.

*In situ* DRIFTS was conducted to study the adsorption behaviors of Ala by using acetic acid (AcOH) as a probe because its simple structure enables clear identification of carboxyl vibrational features. Previous studies have validated AcOH as a reliable model to represent the adsorption and activation of the –COOH group in amino acids.^13,45^ For the Pt/TiO_2_ catalyst, only weak vibrational bands are observed after exposure to AcOH (Fig. 4e), including a weak C=O stretching vibration at 1730 cm^−1^ (*ν*(C=O)), asymmetric and symmetric carboxylate stretching modes at 1645 and 1510 cm^−1^ (*ν*_as_(COO) and *ν*_s_(COO)), respectively, and a methyl bending mode at 1448 cm^−1^ (*δ*(CH_3_)). Although the characteristic bands of adsorbed AcOH can be identified on the Pt/TiO_2_ catalyst, their intensities are relatively weak and decay rapidly within 30 min under Ar flow, indicating weak and reversible adsorption. In contrast, the spectra of the 4-Pt–MoO_3_ catalyst exhibit significantly stronger and more persistent vibrational features. Intense bands at 1645 and 1510 cm^−1^ corresponding to *ν*_as_(COO) and *ν*_s_(COO), respectively, suggest the formation of stable carboxylate species. The C=O stretching band at 1730 cm^−1^ is also more pronounced and remains detectable after prolonged Ar purge, along with a clearly resolved *δ*(CH_3_) signal at 1448 cm^−1^.^13,45^ These vibrational features reflect the coexistence of both molecular and dissociative adsorption states, in contrast to the weak, transient interactions observed on Pt/TiO_2_, indicating MoO_3_ as the anchoring site for polar substrates to stabilize key reaction intermediates.

Mechanistic studies based on DFT calculations, H_2_-TPD, and *in situ* AcOH-DRIFTS reveal that Pt acts as the primary site for H_2_ dissociation, whereas MoO_3_ preferentially serves as the anchoring site for polar functional groups, such as the carboxyl. The observed volcano-shaped trend between activity and the Pt/Mo ratio reflects the need to balance these two complementary functionalities. At an optimal composition (4-Pt–MoO_3_), the rates of hydrogen activation and substrate adsorption are well-matched, resulting in the highest activity and conversion. In contrast, insufficient or excessive MoO_3_ disrupts this balance, either limiting substrate anchoring or suppressing hydrogen activation, thereby reducing overall catalytic efficiency.

### Thermal treatment governed Pt–MoO_3_ synergy for hydrogenation

**Fig. 5:**
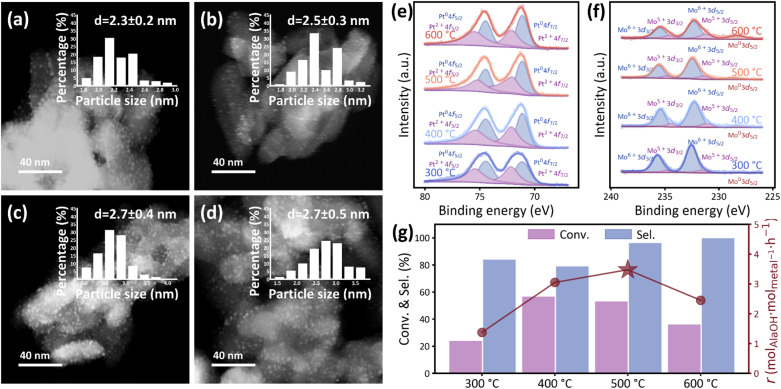
HAADF-STEM images and corresponding particle size distributions of 4-Pt–MoO_3_ reduced at (a) 300, (b) 400, (c) 500 and (d) 600 °C. (e) Pt 4f and (f) Mo 3d XPS spectra of 4-Pt–MoO_3_ catalysts reduced at various temperatures. (g) Catalytic performance of 4-Pt–MoO_3_ catalysts reduced at various temperatures.

The 4-Pt–MoO_3_ catalyst was further reduced at different temperatures ranging from 300 to 600 °C to tune the synergy between Pt and MoO_3_ for the Ala hydrogenation. The morphological characteristics of the 4-Pt–MoO_3_ catalyst reduced at different temperatures were examined by HAADF-STEM imaging. The nanoparticles are uniformly dispersed on TiO_2_ across all the samples, with no signs of significant aggregation, even after high-temperature reduction (Fig. 5a–d). Particle size distributions were obtained by analyzing over 200 individual nanoparticles in each sample. The average particle sizes for the samples reduced at 300, 400, 500 and 600 °C are 2.3 ± 0.2, 2.5 ± 0.3, 2.7 ± 0.4 and 2.7 ± 0.5 nm, respectively, indicating similar morphologies, and therefore, particle size effects can be excluded as the primary factor influencing catalytic behavior. In addition, XRD patterns only show the diffraction peaks of rutile TiO_2_ (JCPDS no. 01-1292) and Pt (JCPDS no. 04-0802) without those assigned to MoO_3_ at all reduction temperatures (Fig. S14). This indicates that Mo species remain amorphous or as highly dispersed MoO_*x*_, with domain sizes below the detection limit of XRD. HRTEM analyses confirm that reduction at 300–600 °C tunes the oxygen vacancy concentration in MoO_*x*_ while preserving the crystal structures of TiO_2_ and Pt (Fig. S5), with MoO_*x*_ lattice fringes (0.22–0.23 nm reported previously) consistently observed.^46^ The influence of thermal reduction on the electronic structure of the catalyst was then evaluated by XPS analysis. As shown in Fig. 5e, the Pt 4f XPS spectra exhibit only slight changes with increasing temperature from 300 to 600 °C. All samples display characteristic Pt^0^ and Pt^2+^ doublet peaks, with the Pt^0^ 4f_7/2_ component located near 71.13 eV. The minor changes in binding energy and relative peak intensity suggest that the oxidation state and electronic environment of Pt remain largely unaffected under the current conditions. Unlike the strong electronic modulation induced by increasing MoO_3_ content (Fig. 3a), the reduction temperature appears insufficient to significantly alter the electron density around Pt sites in the fixed Pt/Mo ratio. In contrast, the Mo 3d XPS spectra reveal a clear evolution in the oxidation state of Mo species with increasing reduction temperature (Fig. 5f). At 300 °C, the spectra are dominated by Mo^6+^ signals, characteristic of stoichiometric MoO_3_. As the temperature increases to 400 °C and beyond, additional peaks corresponding to partially reduced Mo^5+^ and Mo^0^ components become significant, indicating thermal reduction of MoO_3_ under a H_2_ atmosphere.

The Mo 3d XPS spectra were deconvoluted to quantify the fractions of Mo^6+^ and Mo^5+^ species that varied with the reduction temperature. Clearly, with the increasing H_2_ reduction temperature, the amount of Mo^6+^ decreases while that of Mo^5+^ increases (Fig. 5f). In addition, the average valence of oxidized Mo species (*v*_avg_) is defined to compare the differences in the electronic structure caused by various reduction temperatures,^47^ and the value of *v*_avg_ decreases with increasing reduction temperatures (Table S1), evidencing the Pt-promoted formation of oxygen vacancies at elevated temperature. This is also confirmed by the EPR tests, which demonstrate that the signal at *g* = 2.003 appears to be stronger with increasing reduction temperatures (Fig. S27). Such reduction behavior is consistent with previous reports of MoO_3_ reducibility under moderate conditions.^37,38^ The emergence of Mo^0^ signals at high temperatures suggests the formation of oxygen-deficient surface species, potentially associated with the generation of surface oxygen vacancies. These vacancies have been proposed as active sites for polar substrate adsorption and could contribute to the observed catalytic performance. It is also worth noting that, unlike the Mo content-dependent XPS trends shown in Fig. 3b, where excessive loading suppressed Pt-induced reduction *via* electronic dilution, the thermal reduction effect observed here is more direct and progressive, driven by temperature rather than compositional changes. Together, these results underscore the redox flexibility of Mo species under reductive conditions and highlight the critical role of thermal treatment in tuning surface electronic states and potential adsorption sites.

The influence of reduction temperature on the catalytic performance of the 4-Pt–MoO_3_ catalyst was further evaluated. As shown in Fig. 5g and S12, thermal treatment at 500 °C affords the best catalytic performance, with a favorable balance among conversion, selectivity and reaction rate. Under this condition, the catalyst achieves a conversion of 53.1% and a selectivity of 96.3%. As the treatment temperature increases beyond 500 °C, the selectivity continues to improve, reaching nearly 100% at 600 °C. However, this comes at the expense of catalytic activity, as evidenced by a marked decline in conversion (to 36.1%) and a prolonged time to reach full conversion (>22 h). This performance drop is likely due to the excessive reduction of MoO_3_, which may disrupt the electronic interactions between Pt and Mo species or lead to blockage of active adsorption sites on the oxide surface. This trend suggests that moderate treatment effectively balances hydrogen activation on Pt and substrate interaction on MoO_3_, preserving the bifunctional synergy critical for efficient hydrogenation. In contrast, under- or over-treatment leads to suboptimal performance, either due to insufficient electronic modification of MoO_3_ or excessive reduction that impairs functionality.

In addition, since the surface TiO_*x*_ species is possible to be present under high-temperature reduction, it is necessary to address the role of such possibly formed species. Thus, XPS tests for Ti 2p orbitals for the catalysts reduced at various temperatures were further performed. As shown in Fig. S15, the Ti 2p_3/2_ XPS peak shifts slightly from 458.38 to 458.33 eV with increasing reduction temperature from 300 to 600 °C. This very slight shift indicates that while the Ti oxidation state becomes marginally more positive at higher reduction temperatures, the extent of reduction remains limited. Such very small fraction of surface TiO_*x*_ species is believed to deliver minor effects on the catalytic performance. The *in situ* AcOH-DRIFTS spectrum of the Pt–MoO_3_/TiO_2_ catalyst exhibits marked vibrational features associated with carboxyl adsorption compared with Pt/TiO_2_, particularly the *ν*(C=O) stretching and the *ν*_as/s_(COO) bands (Fig. 4e). Furthermore, the Pt/TiO_2_ catalyst exhibits only negligible Ala conversion, underscoring that neither metallic Pt sites nor the limited TiO_*x*_ species generated on TiO_2_ provide sufficient carboxyl adsorption or activation. Upon introducing MoO_3_, both the conversion and the intrinsic reaction rate increase markedly (Fig. 4a). These results unveil that MoO_3_ substantially enhances the adsorption and activation of the carboxyl group, whereas the contribution of TiO_*x*_ remains relatively limited.

Considering that the presence of water as the solvent can decrease the amount of oxygen vacancies in the catalyst,^48^ the cycle-tests were further performed for the optimal catalyst (*i.e.*, 4-Pt–MoO_3_ reduced at 500 °C) to address the catalytic stability. The tests across five consecutive batch runs (4 MPa H_2_, 95 °C, 8 h) reveal that AlaOH yield declines monotonically from 50% to 21% without H_2_ regeneration (Fig. S13). XPS tests for the catalyst after the reaction reveal a slight increase in the valency, and the EPR spectra show a marked attenuation of the signal at *g* = 2.003 assigned to the Mo^5+^ oxygen vacancy after the reaction (Fig. S16 and S26).^49^ These results indicate the decrease of oxygen vacancies in the catalyst after the reaction. In contrast, the initial activity could be recovered when a regeneration under hydrogen at 500 °C was performed between each run, which can be explained by the enriched oxygen vacancies by high-temperature reduction. The contrasting stability results also verify that the hydrogenation of l-alanine follows the reverse Mars–van Krevelen mechanism as schematically shown in Fig. S8.^50–53^

## 4 Conclusions

In summary, a series of bifunctional Pt–MoO_3_/TiO_2_ catalysts were systematically investigated to uncover the structural and electronic origins of their performance in alanine hydrogenation. Advanced characterization techniques, including HAADF-STEM, HRTEM, XPS, and XANES, revealed that the intimate contact between Pt and MoO_3_ induces electronic interactions, characterized by partial electron transfer from Pt to MoO_3_. The H_2_-TPD analysis combined with DFT calculations and *in situ* AcOH-DRIFTS tests support a bifunctional mechanism, wherein Pt activates hydrogen while MoO_3_ facilitates substrate adsorption and stabilization. Catalytic testing demonstrated a volcano-type dependence on the Pt/Mo ratio, with the 4-Pt–MoO_3_ catalyst achieving the highest conversion and selectivity. Further tuning *via* thermal reduction treatments showed that moderate reduction at 500 °C optimally balances the redox state of MoO_3_ without compromising Pt dispersion, leading to enhanced hydrogenation performance. In contrast, excessive reduction at higher temperatures disrupted the delicate synergy and diminished catalytic activity. The insights gained here offer valuable guidance for the rational design of metal–oxide bifunctional systems in selective hydrogenation and even other structure-sensitive catalytic transformations.

## Author contributions

Conceptualization: XZ, XD, and YC. Supervision: XZ, XD, and YC. Funding acquisition: XZ, XD, and YC. Projection administration: YC. Resources: XZ, XD, and YC. Methodology: YJ, RS, XG, and NA. Investigation: YJ, RS, JM, and NZ. Data curation: YJ and RS. Formal analysis: YJ, RS, and NZ. Software: RS and NZ. Visualization: YJ, RS, and XG. Writing – original draft: YJ and RS. Writing – review & editing: all authors.

## Conflicts of interest

There are no conflicts to declare.

## Supplementary Material

SC-OLF-D5SC05792B-s001

## Data Availability

The data supporting this article have been included as part of the supplementary information (SI). Supplementary information: detailed experimental procedures, catalyst synthesis and calculation model, additional characterisation (XRD, XPS, EPR, TEM), supplementary figures and tables underlying the main results. See DOI: https://doi.org/10.1039/d5sc05792b.
