# Supplementary material for: Insights into bifunctional active sites of Pt–MoO3/TiO2 catalysts enabling selective hydrogenation of an amino acid
Source: Chem Sci. 2025 Oct 9;16(46):21897–907. doi: 10.1039/d5sc05792b (PMC12536722; doi:10.1039/d5sc05792b)
Supplement: SC-016-D5SC05792B-s001 [file SC-016-D5SC05792B-s001.pdf]

Supplementary Information (SI) for Chemical Science.
This journal is © The Royal Society of Chemistry 2025

Supporting Information

Insights into bifunctional active sites of Pt-MoO_3/TiO_2 catalyst enabling selective hydrogenation of amino acid

Yundao Jing,[#,a] Xiaohu Ge,[#,a] Rui Song,[a] Ningchao Zhu,[a] Jinquan Ming,[a] Nihong An,[b] Yueqiang Cao,*[a] Gang Qian,[a] Xuezhi Duan*[a] and Xinggui Zhou[a]

[a.] *State Key Laboratory of Chemical Engineering and Low-Carbon Technology, School of Chemical Engineering, East China University of Science and Technology, 130 Meilong Road, Shanghai 200237, China. Email: yqcao@ecust.edu.cn; xzduan@ecust.edu.cn*

[b] *Sino-Platinum Industrial Catalyst (Yunnan) Co., Ltd., 988 Keji Road, Kunming 650106, Yunnan, China.*

[#] *These authors contributed equally to this work.*

1. Experimental Section

Preparation of TiO_2

Commercial rutile-type titanium dioxide (TiO_2, >99%, *$P4_2/mnm$*, Aladdin, China) was used as the support due to its high thermal stability and well-defined crystal structure. Prior to impregnation, TiO_2 powder was pretreated in air at 500 °C for 3 h in a muffle furnace to remove surface impurities, moisture and residual organics, thereby enhancing crystallinity and surface area.

Preparation of Pt/TiO_2

The pore volume of the pre-calcined TiO_2 was measured by water adsorption. An aqueous solution of chloroplatinic acid ($H_2PtCl_6 \cdot 6H_2O$, >99.5%, Macklin, China) was impregnated into the TiO_2 via incipient wetness method. After aging at room temperature, the sample was dried, ground and mildly calcined to remove residual water and stabilize the Pt species.

Preparation of MoO_3/TiO_2

A 30 wt% MoO_3/TiO_2 catalyst was synthesized by impregnating 0.7 g of ammonium molybdate ([$(NH_4)_6Mo_7O_{24} \cdot 4H_2O$], >99.5%, Macklin, China) into 0.37 g of as-prepared TiO_2 with 100 mL deionized water. The mixture was stirred at 50 °C until complete evaporation, followed by drying at 100 °C for 12 h, grinding and calcination in air at 500 °C for 3 h.

Preparation of Pt-MoO_3/TiO_2

The Pt-MoO_3/TiO_2 was prepared through a sequential incipient wetness impregnation. The pore volume of the preformed Pt/TiO_2 was measured, and an aqueous solution of [$(NH_4)_6Mo_7O_{24} \cdot 4H_2O$] was impregnated accordingly. The sample was aged, dried, ground and calcined in air at 500 °C for 3 h. Prior to catalytic testing, the catalyst was reduced under H_2 in a tubular furnace to activate Pt sites and generate oxygen vacancies in MoO_3.

X-ray Photoelectron Spectroscopy (XPS)

XPS measurements were performed on a Thermo Fisher ESCALAB 250Xi spectrometer using Al Kα radiation source (1486.6 eV) at 15 kV. Spectra were analyzed with Thermo Advantage software for peak assignment and deconvolution. All binding energies were calibrated to the C 1*s* peak at 284.8 eV to correct for charging effects.

Transmission Electron Microscopy (TEM)

TEM analysis was performed on a JEOL F200 field emission transmission electron microscope operated at 200 kV. Elemental mapping and compositional analysis were performed using an

Oxford X-MaxN 80T EDS system. High-angle annular dark-field scanning transmission electron microscopy (HAADF-STEM) was employed to observe the particle size and distribution, with size distribution determined by measuring ~200 particles. High-resolution transmission electron microscopy (HRTEM) was further applied to investigate the lattice fringes and nanoparticles morphology, with a point-to-point resolution of 0.23 nm and a line resolution of 0.14 nm. Prior to TEM analysis, the catalysts were ultrasonically dispersed in ethanol for 15 min, and 5 μL of the suspension was dropped onto a copper grid and dried under an infrared lamp.

Hydrogen Temperature-Programmed Desorption (H_2-TPD)

H_2-TPD was conducted on a JWGB JW-HX100 chemisorption analyzer equipped with a thermal conductivity detector (TCD) to investigate hydrogen adsorption and desorption behavior. Approximately 50 mg of catalyst was loaded into a U-shaped quartz tube and purged with 50 sccm 10vol% H_2/Ar at 500 °C for reduction, then cooled to room temperature and exposed to same gas for 1h. After purging with Ar for 1 h to remove physisorbed hydrogen, the sample was heated to 400 °C at 10 °C·min^{-1} and held for 30 min. The desorbed hydrogen was detected by TCD to evaluate the strength and amount of chemisorbed species.

X-ray Absorption Spectroscopy (XAS)

XAS was employed at Pt L_3-edge (11564 eV) on beamline BL16U1 at the Shanghai Synchrotron Radiation Facility (SSRF) to probe the local electronic structure and coordination environment of Pt. Pt foil and PtO_2 standards were recorded in transmission mode for energy calibration and phase-shift reference, while the catalyst samples were measured in fluorescence mode. The acquired spectra were pre-processed and analyzed using Athena and fitted using Artemis, both part of the Demeter software package.[1] Wavelet transform (WT) analysis was performed using the Larch Python library.[2]

Electron Paramagnetic Resonance (EPR)

X-band continuous-wave EPR spectra were recorded on a Bruker A300 spectrometer. Unless otherwise noted, measurements were performed at ambient temperature with a center field of 3500 G. sweep width 200 G, sweep time 30 s, microwave power 19.45 mW, field-modulation amplitude 1.0 G, and conversion time 40 ms. The microwave frequency was measured for each spectrum (representative value v=9.8525 GHz).

***In situ* Diffuse Reflectance Infrared Fourier Transform Spectroscopy (*in situ* DRIFTS)**

In situ DRIFTS using acetic acid (AcOH) as a probe molecule was conducted to study the adsorption behavior of carboxyl-containing species on the catalyst surface. Spectra were collected on a Thermo Fisher Nicolet iS20 FTIR spectrometer equipped with a liquid-nitrogen-cooled MCT detector. The catalyst was loaded into a Harrick Praying Mantis DRIFTS cell, pretreated under 50 sccm H_2 at 500 °C for 1h, and then cooled to room temperature under Ar. After a 30 min Ar purge, a background spectrum was recorded. AcOH vapor was introduced by bubbling high-purity Ar through liquid AcOH in a saturator. Upon reaching adsorption equilibrium, the AcOH flow was stopped and the cell was purged with Ar to remove physisorbed species. Differential spectra were obtained by subtracting the background to identify characteristic vibrational features of AcOH adsorption.[3,4]

Evaluation of Catalytic Performance for L-Alanine Hydrogenation

L-alanine (Ala) hydrogenation was evaluated in a 100 mL Teflon-lined stainless-steel autoclave (YZMRS-650). Aqueous Ala solution was prepared in ultrapure water and adjusted the desired concentration. Typically, 30 mL of this Ala solution, catalyst, and 85 wt% phosphoric acid (as a pH regulator) were added to the reactor. After purging with Ar and H_2, the reactor was pressurized with H_2 and heated to the target temperature under stirring.[5]

Product Analysis

After the reaction, 1 mL of upper phase was sampled, filtered, and analyzed by ultra-performance liquid chromatography (UPLC, Waters Acquity) equipped with a ZORBAX SB-C18 column (4.6 mm × 150 mm, 5 µm), and photodiode array (PDA) detector. Due to the weak UV absorbance of Ala and L-alaninol (AlaOH), samples were derivatized with phenyl isothiocyanate (PITC) and detected at 254 nm.[5,6] Quantification was carried out using the external-standard method. The mobile phase consisted of 0.025 mol·L^{-1} sodium acetate solution, acetonitrile (ACN) and methanol (75:15:10, v/v/v). Calibration curves were obtained from derivatized standards. Reaction samples were treated using the same protocol to ensure consistency.

For derivatization, PITC and triethylamine (TEA) were dissolved in ACN to obtain a 0.1 mol·L^{-1} PITC/ACN and 1.0 mol·L^{-1} TEA/ACN solutions, respectively. A 0.1 mol·L^{-1} HCl aqueous solution was used to acidify the sample. In a typical procedure, 100 µL of standard or reaction sample was diluted with 4.45 mL HCl solution, followed by mixing 200 µL of the diluted solution with 1.0 mL TEA/CAN and 100 µL PITC/CAN. The mixture was reacted at room temperature for 1h, then

extracted with 400 µL n-hexane. The organic phase was discarded, and the aqueous layer was diluted 4-fold with water and filtered before UPLC injection.

The derivatized Ala and AlaOH eluted at ~3.5 min and ~10 min, respectively, with symmetric peak and good resolution. Conversion and selectivity were calculated as follows:

$$Conversion_{Ala}(\%) = \frac{C_{0,\,Ala} - C_{t,Ala}}{C_{0,\,Ala}} \times 100$$

$$Selectivity_{AlaOH}(\%) = \frac{n_{AlaOH}}{\sum_i n_i} \times 100$$

where $C_{0,\,Ala}$ and $C_{t,Ala}$ are the initial and final concentrations of Ala, and n_i denotes the molar amounts of each detected products. All quantitative analyses were performed at least in duplicate to ensure reproducibility.

The rate of alaninol formation r (in $mol_{AlaOH} \cdot mol_{Pt+Mo}^{-1} \cdot h^{-1}$) was calculated based on the following equation:

$$r = \frac{n_{AlaOH}}{t \times n_{active\ site}}$$

where n_{AlaOH} represents the amount of alaninol formed within a given reaction the time t, and $n_{active\ site}$ denotes the number of moles of surface-exposed active sites on catalyst.

2. Density Functional Theory (DFT) Calculations

Computational Details

DFT calculations were performed using the Vienna Ab initio Simulation Package (VASP) with the projector augmented-wave (PAW) method and the Perdew-Burke-Ernzerhof (PBE) functional within the generalized gradient approximation (GGA) framework.[7,8] A plane-wave cutoff energy of 450 eV and an augmentation charge cutoff of 650 eV were applied. Geometry optimizations were performed using the conjugate-gradient algorithm with an energy convergence threshold of 1×10^{-5} eV and a maximum residual force criterion of 0.05 eV·$Å^{-1}$. Brillouin zone sampling used a 3×3×1 Monkhorst-Pack *k*-point mesh appropriate for surface slab models.

Surface Models and Adsorption Calculations

Slab models of Pt(111) and MoO_3(060) were constructed as (3×3) and (4×4) supercells with four atomic layers, respectively. During surface relaxation, the bottom two layers of atoms were fixed and upper layers were fully relaxed. A 20 Å vacuum region was applied along the z-direction to avoid interlayer interactions. For MoO_3, a DFT+U correction with U-J=6.3 eV was applied to account for Mo 4*d* electron correlation.[9] Oxygen-defective MoO_x(060) surface was generated by removing a surface O atom from MoO_3(060).

Adsorption Energy Calculations

The adsorption energy E_{ads} of adsorbates on various surfaces was calculated using:

$$E_{ads} = E_{adsorbate/surface} - E_{adsorbate} - E_{surface}$$

Where $E_{adsorbate/surface}$ is the total energy of the adsorbate-surface system, $E_{adsorbate}$ is the energy of the isolated molecule, and $E_{surface}$ is the energy of the clean surface.

Transition-State Search

Transition states were identified using the nudged elastic band (NEB) method with climbing-image refinement to accurately locate the saddle point.[10] An improved tangent scheme was adopted to allow automatic adjustment of spring forces between images, enhancing the stability and convergence of the calculation. Four intermediate configurations were generated between the initial and final states to map the minimum energy pathway.

3. Supplementary Figures and Tables

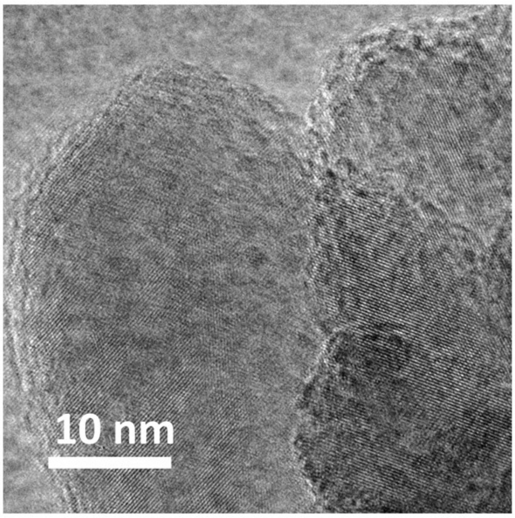

Fig. S1. High-resolution TEM image of the 1-Pt-MoO_3 catalyst showing well-defined lattice fringes and particle morphology.

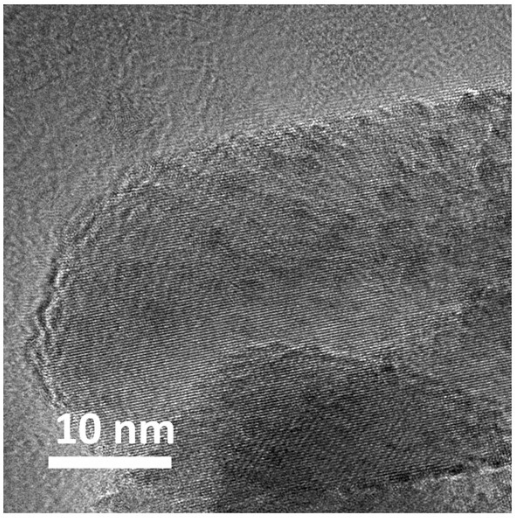

Fig. S2. High-resolution TEM image of the 2-Pt-MoO_3 catalyst showing well-defined lattice fringes and particle morphology.

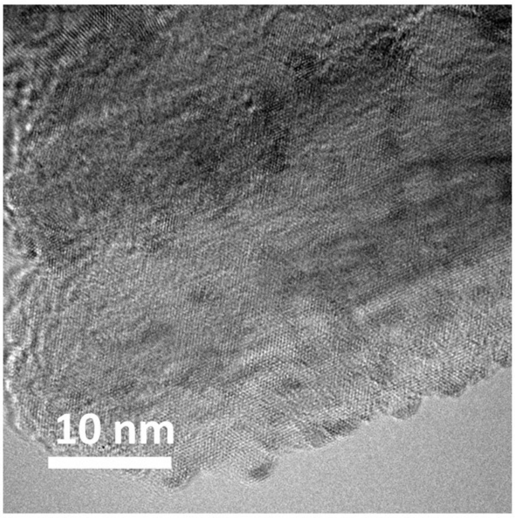

Fig. S3. High-resolution TEM image of the 4-Pt-MoO_3 catalyst showing well-defined lattice fringes and particle morphology.

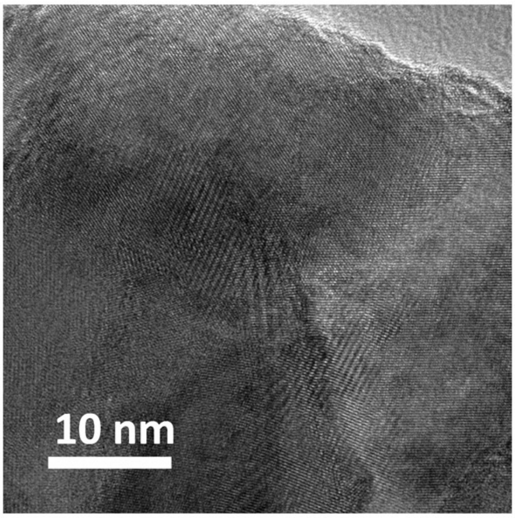

Fig. S4. High-resolution TEM image of the 6-Pt-MoO_3 catalyst showing well-defined lattice fringes and particle morphology.

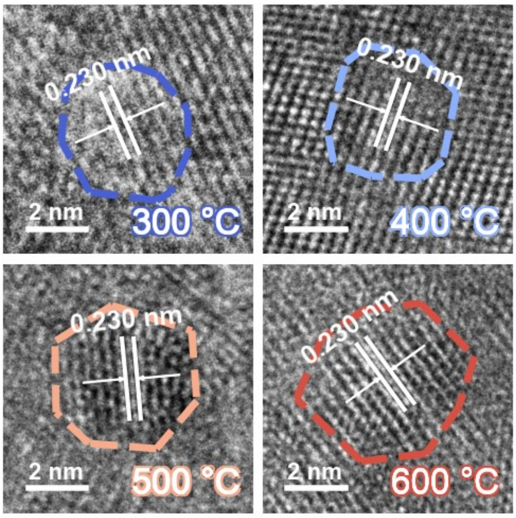

Fig. S5. HRTEM images of Pt-MoO_3/TiO_2 reduced at 300, 400, 500, and 600 °C.

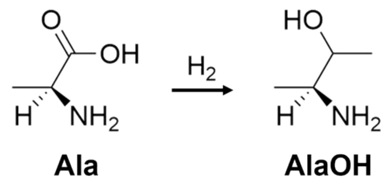

Fig. S6. Reaction pathway of L-alanine (Ala) hydrogenation to L-alaninol (AlaOH).

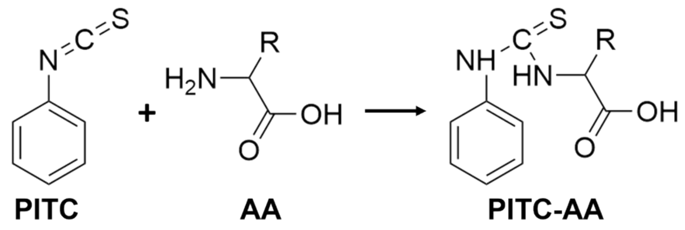

Fig. S7. Schematic illustration of the derivatization of amino acids (AA) with phenyl isothiocyanate (PITC) to form PITC derivatives (PITC-AA).

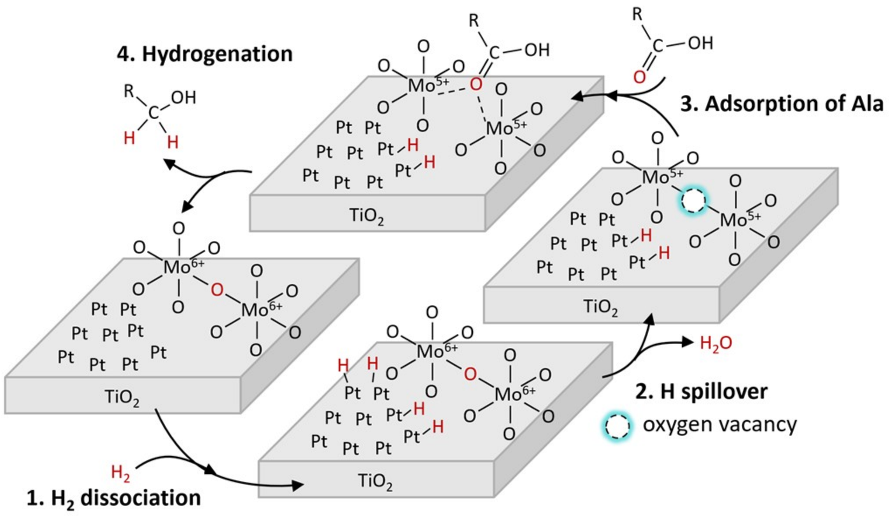

Fig. S8. Proposed HDO mechanism over the Pt-MoO_3/TiO_2 bifunctional catalyst. (1) H_2 dissociation on Pt sites. (2) H-spillover to Mo-O units of the MoO_3, inducing partial reduction (Mo^{6+} to Mo^{5+}) and creating oxygen vacancies. (3) Adsorption/activation of the Ala at Mo^{5+} sites. (4) Hydrogenation assisted by H supplied from Pt, yielding the reduced product. Scheme redrawn and adapted from ref. 51 with minor modifications.

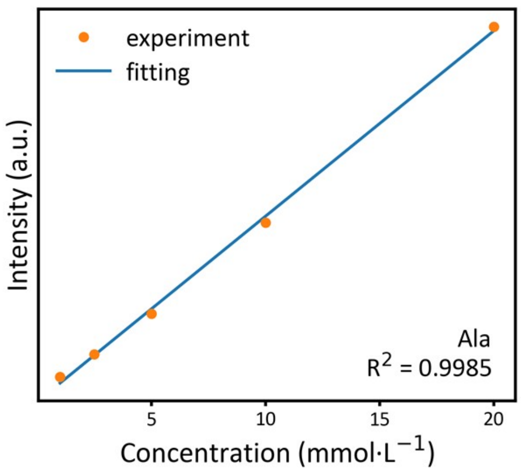

Fig. S9. Standard calibration curve of L-alanine (Ala) obtained by UPLC analysis, showing the linear relationship between concentration and peak intensity (R^2=0.9985).

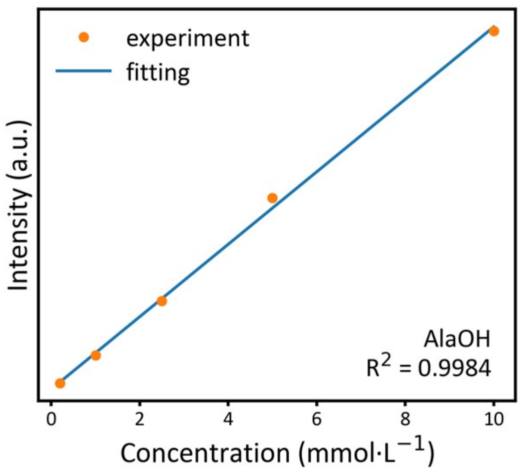

Fig. S10. Standard calibration curve of L-alaninol (AlaOH) obtained by UPLC analysis, showing the linear relationship between concentration and peak intensity (R^2=0.9984).

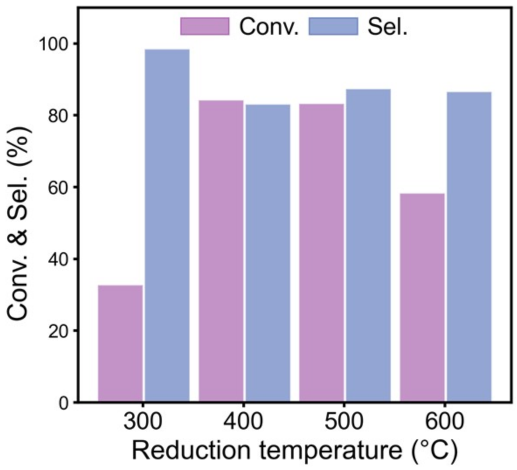

Fig. S11. Hydrogenation performance of 4-Pt-MoO_3 reduced at different temperatures for 5 mmol·L^{-1} (Ala).

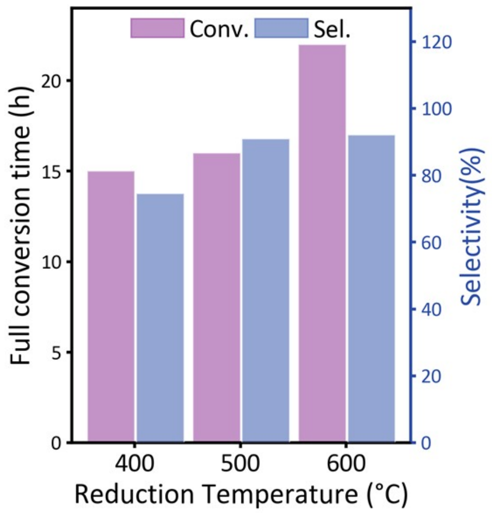

Fig. S12. Full conversion time and corresponding selectivity for 10 mmol·L^{-1} Ala of 4-Pt-MoO_3 catalysts reduced at different temperatures.

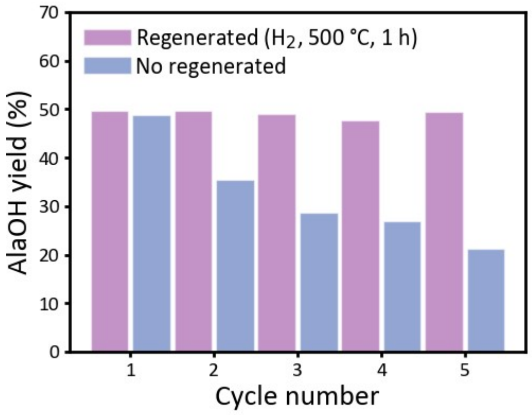

Fig. S13. AlaOH yield versus cycle number for catalysts regenerated with H_2 at 500 °C for 1 h prior to each run (purple) and reused without regeneration (blue).

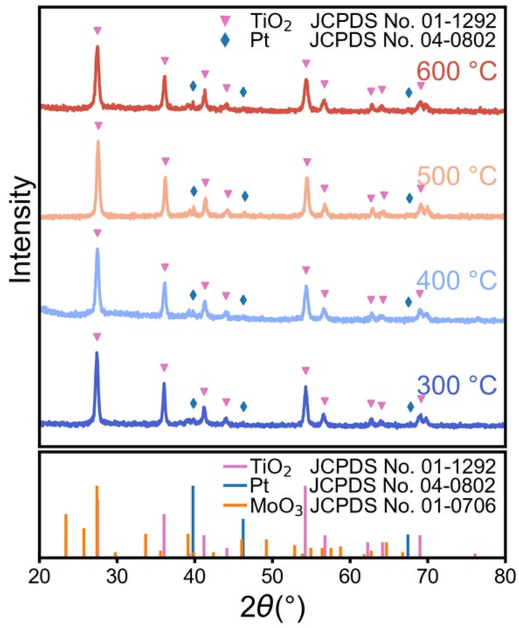

Fig. S14. XRD patterns of Pt-MoO_3/TiO_2 reduced at 300, 400, 500, and 600 °C.

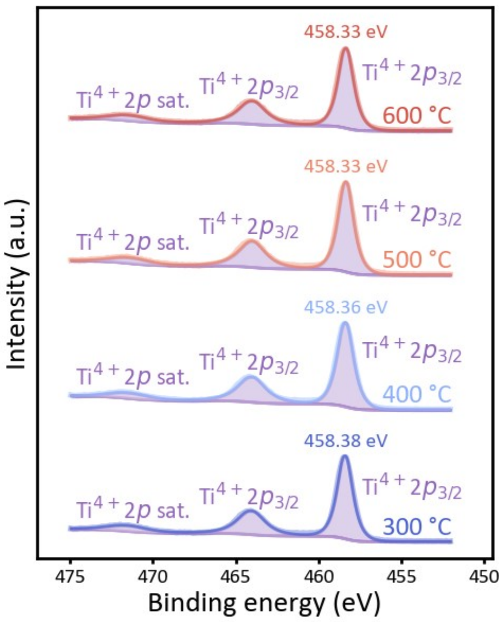

Fig. S15. Ti 2*p* XPS spectra of 4-Pt-MoO_3 reduced at 300, 400, 500, and 600 °C.

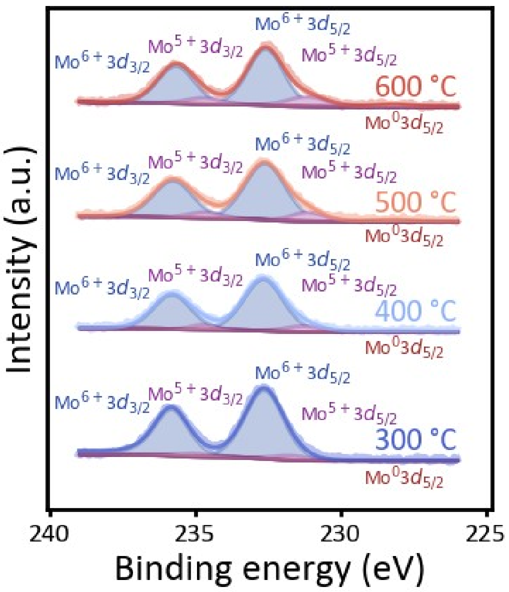

Fig. S16. Mo 3*d* XPS spectra of 4-Pt-MoO_3 catalysts after reaction at 95 °C under 4 MPa H_2 for 8 h; catalysts were reduced at the indicated temperatures prior to reaction.

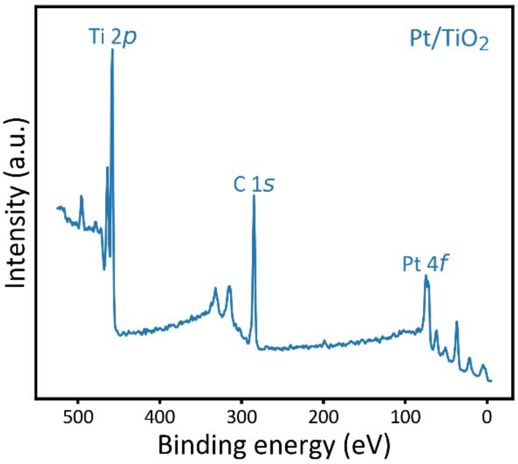

Fig. S17. XPS survey spectrum of Pt/TiO_2 catalyst, showing characteristic peaks of Ti 2*p*, C 1*s* and Pt 4*f*.

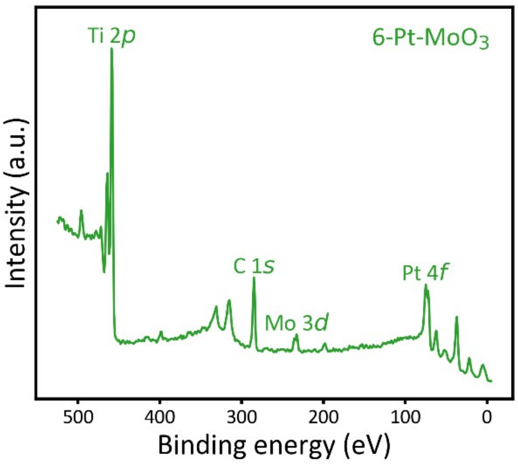

Fig. S18. XPS survey spectrum of 6-Pt-MoO_3 catalyst, showing characteristic peaks of Ti 2*p*, C 1*s*, Mo 3*d* and Pt 4*f*.

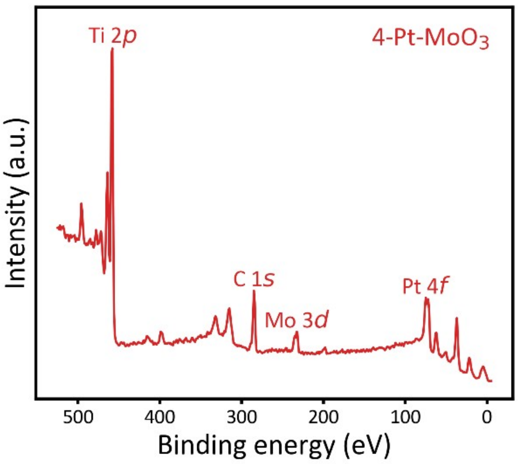

Fig. S19. XPS survey spectrum of 4-Pt-MoO_3 catalyst, showing characteristic peaks of Ti 2*p*, C 1*s*, Mo 3*d* and Pt 4*f*.

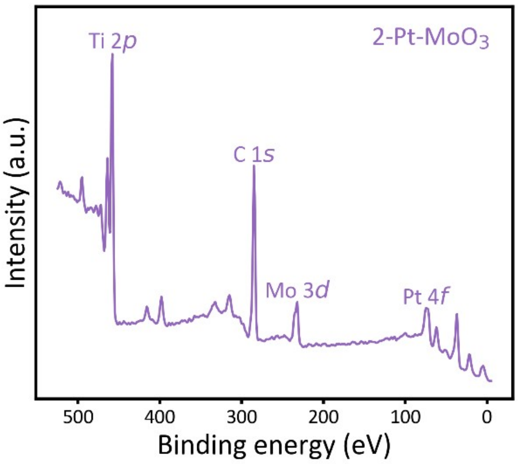

Fig. S20. XPS survey spectrum of 2-Pt-MoO_3 catalyst, showing characteristic peaks of Ti 2*p*, C 1*s*, Mo 3*d* and Pt 4*f*.

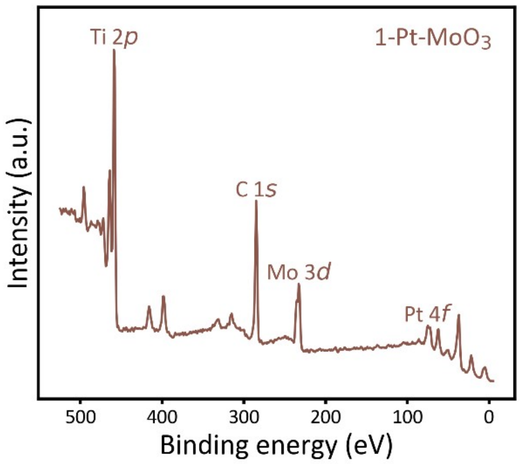

Fig. S21. XPS survey spectrum of 1-Pt-MoO_3 catalyst, showing characteristic peaks of Ti 2*p*, C 1*s*, Mo 3*d* and Pt 4*f*.

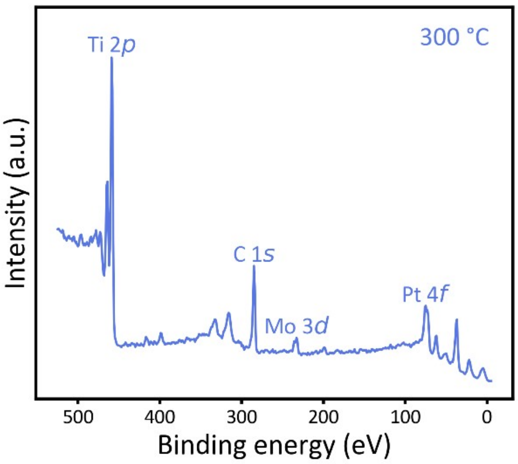

Fig. S22. XPS survey spectrum of 4-Pt-MoO_3 catalyst reduced at 300 °C, showing characteristic peaks of Ti 2*p*, C 1*s*, Mo 3*d* and Pt 4*f*.

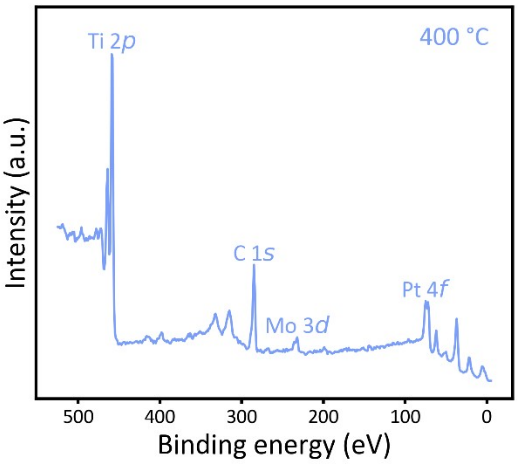

Fig. S23. XPS survey spectrum of 4-Pt-MoO_3 catalyst reduced at 400 °C, showing characteristic peaks of Ti 2*p*, C 1*s*, Mo 3*d* and Pt 4*f*.

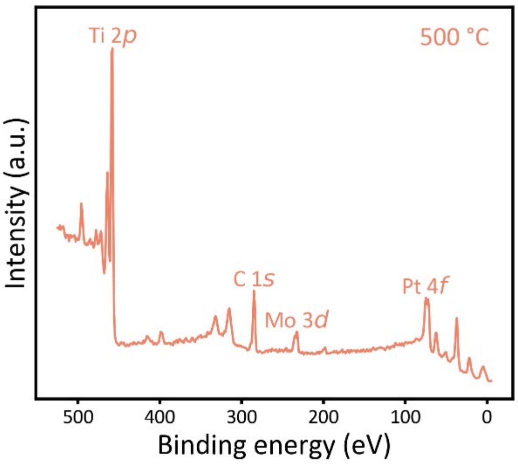

Fig. S24. XPS survey spectrum of 4-Pt-MoO_3 catalyst reduced at 500 °C, showing characteristic peaks of Ti 2*p*, C 1*s*, Mo 3*d* and Pt 4*f*.

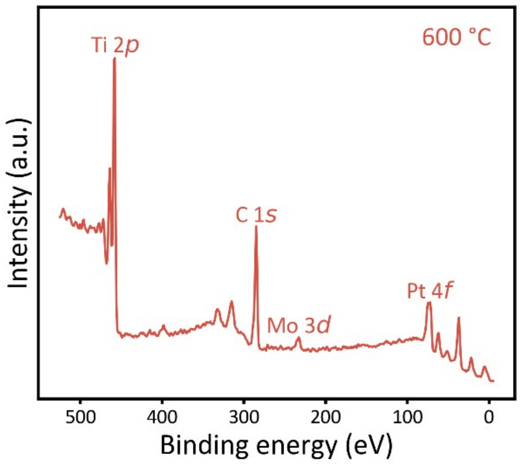

Fig. S25. XPS survey spectrum of 4-Pt-MoO_3 catalyst reduced at 600 °C, showing characteristic peaks of Ti 2*p*, C 1*s*, Mo 3*d* and Pt 4*f*.

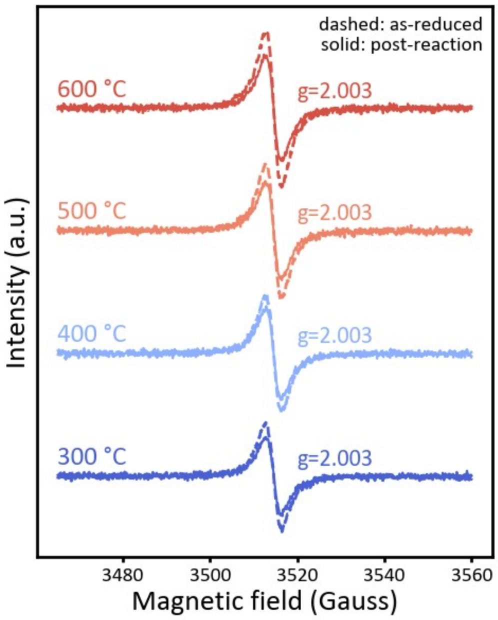

Fig. S26. The EPR spectra of 4-Pt-MoO_3 catalysts reduced at the indicated temperatures, collected before reaction (dashed, as-reduced) and after reaction (solid, 95 °C, 4 MPa H_2, 8 h).

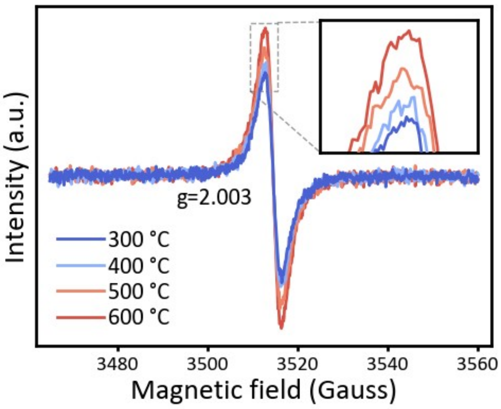

Fig. S27. The EPR spectra of 4-Pt-MoO_3 catalysts reduced at various temperatures.

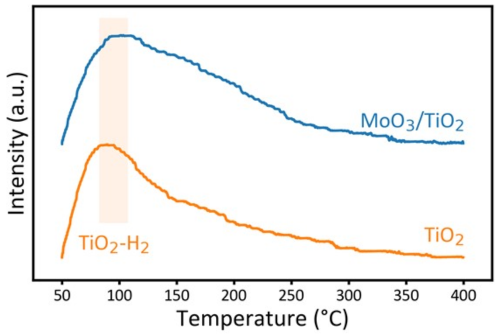

Fig. S28. H_2-TPD profiles of TiO_2 and MoO_3/TiO_2.

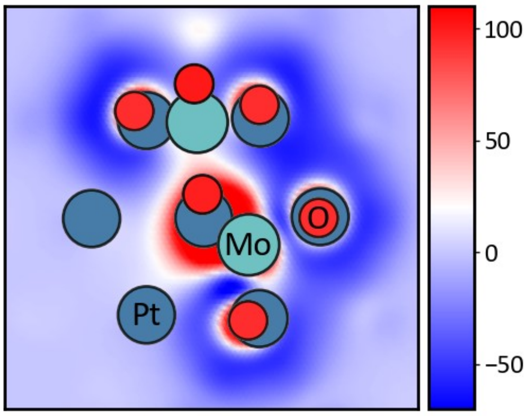

Fig. S29. Charge-density difference map for a MoO_3 cluster on Pt. Red/blue denote electron accumulation/depletion, where units of the color bars are e·$Å^{-3}$.

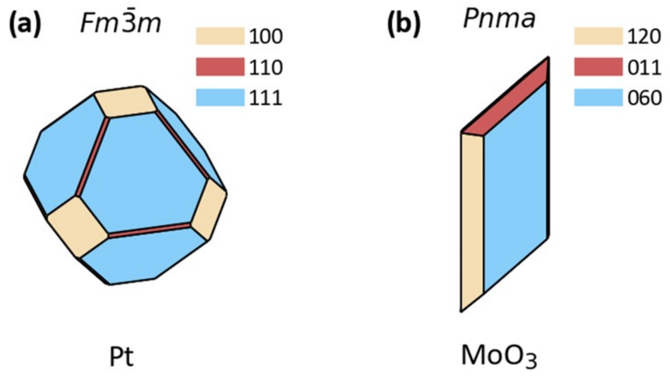

Fig. S30. Wulff crystals for (a) Pt and (b) MoO_3, where the crystal facets denoted by Miller indices are shown by different colors.

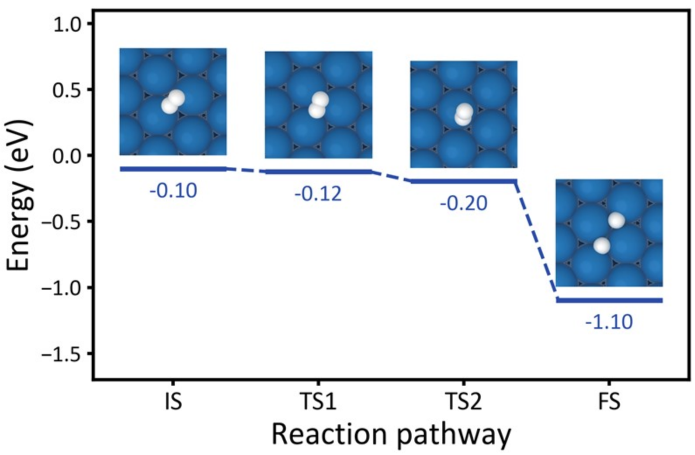

Fig. S31. DFT-calculated energy profile of H_2 adsorption and dissociation on the Pt(111) surface, including initial state (IS), transition state (TS1 and TS2) and final state (FS).

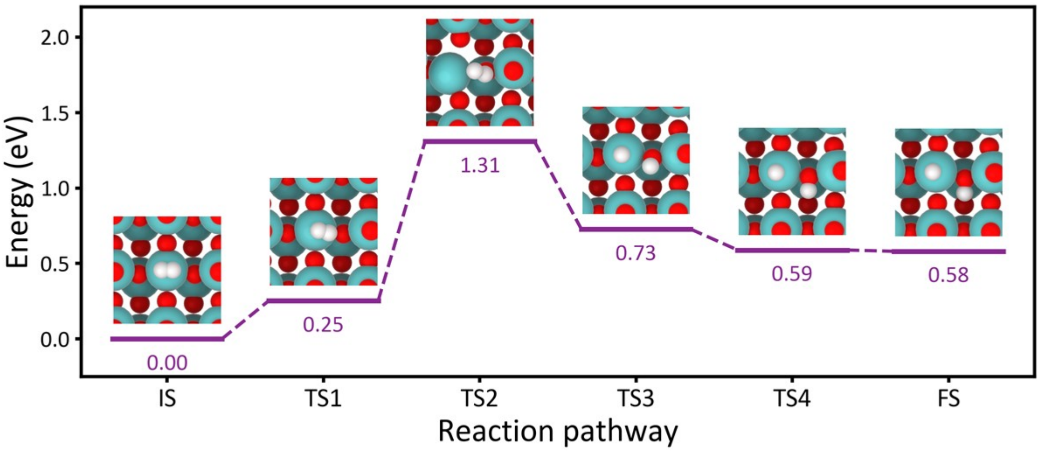

Fig. S32. DFT-calculated energy profile of H_2 adsorption and dissociation on the MoO_x(060) surface, including initial state (IS), transition state (TS1,TS2, TS3 and TS4) and final state (FS).

Table S1. Mo oxidation-state composition of 4-Pt-MoO_3 after H_2 reduction at the indicated temperatures derived from Mo 3*d* XPS spectra.

Reduction temperature (°C)	Mo^{6+} fraction	Mo^{5+} fraction	Average Mo valence, v_{avg}
300	0.90	0.10	5.90
400	0.87	0.13	5.87
500	0.85	0.15	5.85
600	0.78	0.22	5.78

Table S2. Mo oxidation-state composition of 4-Pt-MoO_3 after catalytic reaction (95 °C, 4 MPa H_2, 8 h), for samples pre-reduced at the indicated temperatures.

Reduction temperature (°C)	Mo^{6+} fraction	Mo^{5+} fraction	Average Mo valence, v_{avg}
300	0.93	0.07	5.93
400	0.91	0.09	5.91
500	0.88	0.12	5.88
600	0.85	0.15	5.85

Table S3. Calculated surface properties of Pt facets used in Wulff construction.

Surface	(100)	(110)	(111)
Energy (eV)	-344.14	-332.49	-165.81
a (Å)	8.324	11.771	8.324
b (Å)	8.324	8.324	8.324
Angle (°)	90	90	120
Area (Å^2)	69.29	97.99	60.00
Number of atoms	54	54	27
γ (eV·Å^{-2})	0.2278	0.2206	0.1837

Table S4. Calculated surface properties of MoO_3 facets used in Wulff construction.

Surface	(101)	(210)	(600)
Energy (eV)	-588.14	-590.50	-308.09
a (Å)	14.759	11.756	11.044
b (Å)	11.044	16.021	7.837
Angle (°)	90	90	90
Area (Å^2)	162.99	188.35	86.56
Number of atoms	96	96	48
γ (eV·Å^{-2})	0.1170	0.0950	0.0292

Table S5. DFT-optimized configurations of Ala adsorbed on Pt(111) surface.

E_{ads} (eV)	-1.85	-1.87	-1.84
Top view	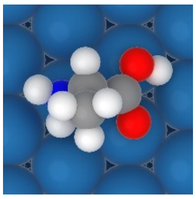	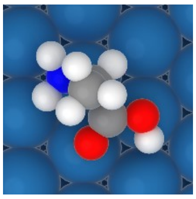	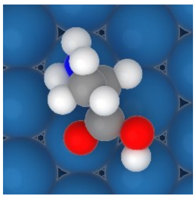
Side view	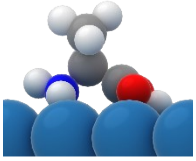	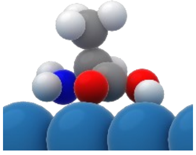	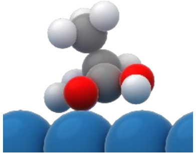

Table S6. DFT-optimized configurations of Ala adsorbed on MoO_x(060) surface.

E_{ads} (eV)	-2.65	-1.64	-1.07
Top view	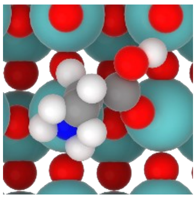	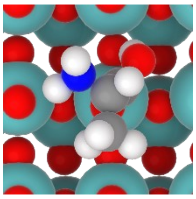	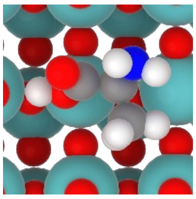
Side view	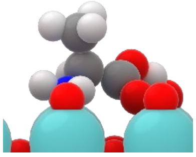	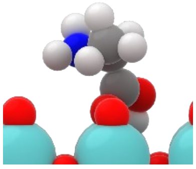	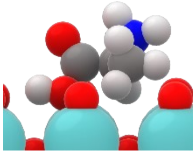

Table S7. DFT-optimized configurations of AlaOH adsorbed on Pt(111) surface.

E_{ads} (eV)	-1.93	-1.94	-1.30
Top view	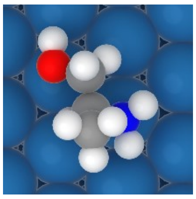	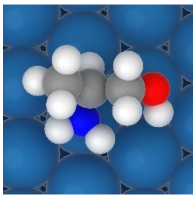	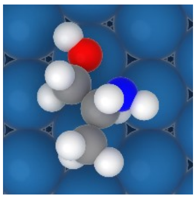
Side view	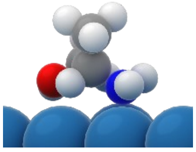	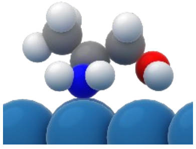	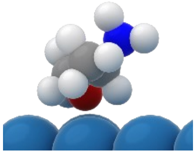

Table S8. DFT-optimized configurations of AlaOH adsorbed on MoO_x(060) surface.

E_{ads} (eV)	-2.88	-1.73	-2.43
Top view	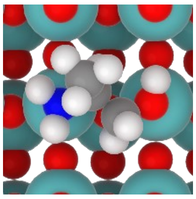	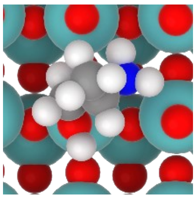	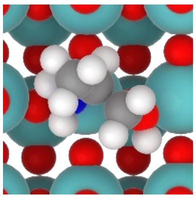
Side view	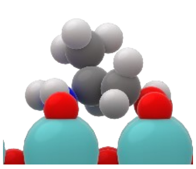	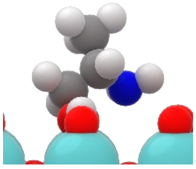	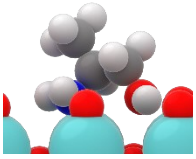

Reference

1 B. Ravel and M. Newville, ATHENA, ARTEMIS, HEPHAESTUS: data analysis for X-ray absorption spectroscopy using IFEFFIT, *J Synchrotron Radiat*, 2005, **12**, 537-541.

2 M. Newville, Larch: An Analysis Package for XAFS and Related Spectroscopies, *Journal of Physics: Conference Series*, 2013, **430**.

3 M. Tamura, R. Tamura, Y. Takeda, Y. Nakagawa and K. Tomishige, Insight into the mechanism of hydrogenation of amino acids to amino alcohols catalyzed by a heterogeneous MoO_x -modified Rh catalyst, *Chemistry*, 2015, **21**, 3097-3107.

4 C. Kaku, S. Suganuma, K. Nakajima, E. Tsuji and N. Katada, Selective Hydrogenation of L-proline to L-prolinol over Al_2O_3-supported Pt-MoO_x Catalyst, *ChemCatChem*, 2022, **14**.

5 R. Song, C. Yao, W. Li, N. An, Y. Shen, N. Fei, X. Ge, Y. Cao, X. Duan and X. Zhou, Kinetic insights into structure sensitivity of Ru catalyzed l-alanine hydrogenation to alaninol, *Reaction Chemistry & Engineering*, 2025, **10**, 135-145.

6 P. Kwanyuen and J. W. Burton, A Modified Amino Acid Analysis Using PITC Derivatization for Soybeans with Accurate Determination of Cysteine and Half-Cystine, *Journal of the American Oil Chemists' Society*, 2009, **87**, 127-132.

7 J. P. Perdew, K. Burke and M. Ernzerhof, Generalized gradient approximation made simple, *Physical review letters*, 1996, **77**, 3865.

8 G. Kresse and J. Furthmüller, Efficient iterative schemes for ab initio total-energy calculations using a plane-wave basis set, *Physical review B*, 1996, **54**, 11169.

9 S. O. Akande, A. Chroneos, M. Vasilopoulou, S. Kennou and U. Schwingenschlögl, Vacancy formation in MoO_3: hybrid density functional theory and photoemission experiments, *Journal of Materials Chemistry C*, 2016, **4**, 9526-9531.

10 G. Henkelman, B. P. Uberuaga and H. Jónsson, A climbing image nudged elastic band method for finding saddle points and minimum energy paths, *The Journal of chemical physics*, 2000, **113**, 9901-9904.
